# Book Reviews

**Published:** 1824-05

**Authors:** 


### CRITICAL ANALYSIS
### OF
### ENGLISH AND FOREIGN LITERATURE
### RELATIVE TO THE VARIOUS BRANCHES OF
### Medical Science.

Quæ laudanda forent, et quæ culpanda, vicissim

Illa, prius, cretâ; mox hæc, carbone, *notamus*.—PERSIUS.

### DIVISION I.

### ENGLISH.

ART. I.—*Transactions of the Medico-Chirurgical Society of Edinburgh; instituted August* 2, 1821. With Plates.—8vo. pp. 697. Edinburgh, 1824.

WE have much pleasure in drawing the attention of our readers to the volume now before us. Rumors have gone abroad, since some of the recent changes in the northern university, that the days of its renown were gone by, and that, like every thing earthly, this great school of medicine had waxed old, even to decay,—that "Troja fuit" was the only fitting emblem to be written over its gates. To what extent these reports may be correct, or whether the worthy burghers of that ancient city displayed absolute wisdom in their arrangements, it would be foreign to our present object to inquire; and indeed we hint at these matters only because we conceive it not improbable that some good may have arisen out of apparent evil, and that a desire of rescuing themselves from the suspicion of degenerating from their immediate predecessors may have had some influence in inducing the contributors to the present volume to show that the *modern Athens* still contains some choice spirits in medical science, as well as in literature.

The papers constituting these Transactions amount in number to twenty-six, and, as might be expected, are of very different degrees of merit. Some have evidently been admitted out of deference to the contributors, (such, for example, are Mr. Russel's, and the last paper in the volume by Dr. Abercrombie, neither of which contain any thing worthy of these gentlemen;) and others apparently to swell the volume. Notwithstanding these circumstances, however, the Transactions contain much valuable matter; so much, indeed, that our readers must excuse us if we restrict ourselves to laying but a part of it before them. At first we had intended to analyse each paper separately; but the limited space we can devote to the article would, if we attempted this, give to it a composition so unconnected,—so entirely made up of shreds and patches, that we have judged it more expedient to select a few papers, of which to give a more extended account.

*An Account of the Appearances observed in the Dissection of two or three Individuals presumed to have perished in the Storm of the 3d, and whose Bodies were discovered in the Vicinity of Leith on the Morning of the 4th November,* 1821; *with some Reflections on the Pathology of the Brain.* By George Kellie, M.D. &c.

Dr. Kellie commences his interesting paper by informing us that, in November 1821, after a tempestuous night, three dead bodies were found in the neighbourhood of Leith, under circumstances which rendered it probable that they had fallen victims to the inclemency of the weather. Two of the bodies were examined by the author and Mr. Cheyne; one was that of a man about forty, the other a female past sixty: both were free from any external blemish. Very little blood flowed on dividing the scalp; the whole of the dura mater in the man was somewhat congested, suffused, and heightened in colour; in the woman these appearances were less distinct; in both the sinuses were loaded with blood, and the veins of the pia mater, together with those between the convolutions, were very turgid. About three ounces of serous fluid were found in the ventricles and basis of the skull, in both; while in the man there was superadded an effusion of a similar nature between the arachnoid and pia mater.

On opening the abdomen, a similar coincidence of appearances was observed. The small intestines, in both subjects, were of a deep red colour, from minute vascular conjestion; in both the stomach and colon were pale; the former having, on its mucous surface, some spots, in the one case of "a coffee-ground colour and ecchymosed appearance," and in the other of a "florid purple." No other deviations from the usual state of the viscera were found in the abdomen, except that in the female the pancreas was of a dark flesh colour.

The night had been very dark, with a heavy gale of wind, and drifting showers of sleet and snow; but it appears that the temperature had not fallen below 34°, and consequently these cases are rather to be regarded as examples of death from exhaustion and torpor, consequent upon continued exposure to the benumbing influence of wet and cold, than of any thing resembling frost-bite.

Another instance is referred to by Dr. Kellie, of an individual who had perished under similar circumstances, and in whom the vessels of the brain were found turgid, together with a serous effusion into the ventricles. It is related by QUELMALZ, and copied into the sixth volume of HALLER's "Disputationes."

These cases seem to have led our author to the inquiry into the circulation of the blood in the brain, which constitutes the main object of his paper; and, in following him through this disquisition, we shall give his opinions in his own words, as far as our limits admit.

"The signs, however, of what is considered compression, were found to exist within the heads of our cases, and of that related by Quelmalz. Congestion, indeed, and the effusion of from three to four ounces of fluid within the head, are appearances commonly considered as indications of compression, and as affording no unsatisfactory explanation of the phenomena of a previous disease, the symptoms of which had been those of disorder or suppression of the functions of the brain. If, on the dissection of a patient who had died of a disease characterised by all the ordinary symptoms of any of the comata, the physician were to discover such appearances as were found in these cases, he would be satisfied, congratulate himself perhaps on the accuracy of his diagnosis, and admire the correspondence between the symptoms of functional derangement, and the lesions discovered in the organs of those functions. In various head-cases, I have certainly seen a less satisfactory concordance between the symptoms and the organic changes discovered on dissection; and many such examples might easily be produced from the recorded experience of other physicians. I am quite aware of the objection, that these indications of what is called compression, discovered in the brain of those who have died from cold, are rather to be regarded as contingent effects than the cause of the apoplexy which terminated in death; or, in other words, that the observed congestion and effusion are the effects of the retarded return of the blood from the head, the consequence of a general immobility of the nervous power induced by the sedative action of cold. The objection has been thus stated by the illustrious Cullen; "With respect, however, to the circumstances which may appear upon the dissection of persons dead of apoplexy, there may be some fallacy in judging, from those circumstances, of the cause of the disease. Whatever takes off or diminishes the mobility of the nervous power, may very much retard the motion of the blood in the vessels of the brain, and that perhaps to the degree of increasing exhalation, or even of occasioning rupture and effusion; so that, in such cases, the marks of compression may appear upon dissection, though the disease had truly depended on causes destroying the mobility of the nervous power.'

"In admitting the force of this objection, I must remark, that the explanation cannot be limited to those cases arising from the action of narcotics and cold on the nervous system, but may, with great truth, be extended to many of Dr. Cullen's cases of apoplexy from compression. In many, for example, where one or more attacks of simple apoplexy have been recovered from, and one at last proves fatal,—where a gouty, paralytic, or epileptic individual, is suddenly taken off by a paroxysm of apoplexy,—where head-aches, vertigo, sickness, and lethargy, have slowly led on to fatal carus or apoplexy,—or when death has been ushered in by hydrocephalic fever,—we may, on dissection, discover congestions and effusions of blood or of serum, which we justly regard as connected, in the order of cause, with the last fatal attack, or with the symptoms of the more advanced disease; but to which we should, I apprehend, err in attributing all the symptoms which marked the approach, or constituted the earlier stages of such cases. I am disposed, indeed, to consider the appearances of congestion of the brain, observed in dissections, as always somewhat questionable and equivocal. It is certain, I think, that the appearances exhibited by the vascular system after death, give no very true or accurate representation of the balance of circulation as carried on during life. During life, the blood is shared, in some proportion or other, between the arterial and venous systems; and, however much the balance may at different times vary between these systems, still the circulating fluid is constantly passing from the one to the other, and must, at every instant of time, be divided between them in such a way, that neither can ever be perfectly depleted or congested at the expence of the other. Not so when life has ceased; for then the arterial is found to be comparatively deprived of, and the venous system to be congested with, blood. This is strikingly true in particular parts, and in none more remarkably than in the brain. In no part of the body, with the exception perhaps of the cavæ and sinus venosus, do we find, on dissection, so much of venous congestion as in the brain: the sinuses of the dura mater are almost always loaded, and the veins at the basis and on the surface of the brain are commonly distended with blood. In some cases this congestion is certainly more remarkable than in others; and often we are enabled to connect this greater than usual congestion with symptoms which, during life, had seemed to predicate such a state. But here, too, we find but little blood in the arteries, and the less, perhaps, the more the veins appear congested. It may therefore be concluded, that the blood, which after death we find congested within one set of vessels in the brain, is just that quantity of blood which was circulated within the head, and at every instant of time distributed, in some proportion or other, between the arteries and veins during life." (p. 94—97.)

With regard to effusions, the same line of argument is adopted; and it is concluded that, when these take place, it is at the expence of a like quantity of the circulating fluid. Indeed, the object to be proved in this part of the paper may be stated in few words:—The head is supposed to be a hollow sphere, of unyielding materials, filled with incompressible contents, so that we cannot force one particle more into it without forcing a corresponding particle out of it, and *vice versa*: and the inference from this is, that we cannot by bleeding diminish the quantity of blood in the brain. These opinions are by no means new, nor indeed does our author advance them as such; having been adopted and illustrated by various distinguished writers, among whom are Monro *secundus*, Cheyne, and Abercrombie. Any one at all accustomed to witness examinations of the brain after death, must have been struck with the great uniformity presented by its blood-vessels, under whatever circumstances death may have occurred, the blood being almost always contained in the veins, so as to distend them to a greater or less extent, and giving rise to the statement, in nineteen cases out of twenty, where an account of the dissection is published, "that the vessels of the brain were *unusually* distended." We have often wondered that it has never struck gentlemen, relating their cases, that what occurs so frequently cannot be *unusual*, whether it be morbid or not: but this *en passant*.

Is it then true, asks Dr. Kellie, and consistent with experience, that we cannot lessen, to any considerable extent, the quantity of blood within the cranium, by arteriotomy or venesection? This is matter of experiment rather than reasoning, and accordingly has been put to this test.

"Having obtained permission from a butcher, I opened the carotid artery of one of his sheep, and the jugular vein of another, in presence of Dr. Duncan, junior, Dr. Anderson, and Dr. Combe. The moment the artery was opened, the blood was projected with great force to a distance of several feet; soon after it flowed more slowly, and per saltum only, and the jet gradually became smaller and smaller. We observed (as Dr. Parry had done) the gradual contraction of the calibre of the artery as the vascular system became emptied, and we saw that it has itself no pulsatory motion, or alternate dilatation and contraction.

"Ten minutes after the carotid had been opened, the breathing was hurried and laborious, and the animal was slightly convulsed. The blood for ten minutes trickled more slowly down the neck; the eye became heavy and listless, and the breathing more and more oppressed; and, at twenty minutes from the commencement of the experiment, there was a general convulsion, and the animal instantly expired.

"When the vein was opened, the blood gushed out in a copious stream, but soon began to issue more slowly. In twelve minutes the sheep was convulsed, and in twenty-one minutes from the time of opening the vein he died. These sheep were immediately fleeced, and cut up in the usual way by the butcher; and the heads were separated, labelled, and set aside for examination on the following morning.

A, The sheep bled from the carotid artery.—The dura mater contained but little blood; the sinuses were full; the pia mater was well injected; and the vessels following the convolutions of the brain were seen full, tortuous, and anastomosing freely; the choroid plexus was well filled with florid blood.

B, The sheep bled from the jugular vein.—The integuments of the head of this sheep were observed to yield more blood, when divided, than the head of the former. The dura mater was rather paler; the larger vessels of the pia mater were well filled, but the minute injection and colouring of the membrane was less remarkable than in the sheep bled from the carotid. The sinuses at the basis cranii were loaded with dark-coloured blood. The choroid plexus was not so well injected, and not so florid as in the first animal.

C, We examined the heads of two sheep, which had been slaughtered for the market by the butcher in the usual way. Blood was found in all the sinuses; several florid red vessels were seen ramifying over the brain and membranes; but these brains were decidedly paler, and we all agreed that they contained less red blood than the brains of the two sheep whom we had bled. They had a more watery and serous aspect, as it were; but there was no palpable effusion of serum on their surface, or within their ventricles.

"Some days after, having obtained three other sheep, one of them was slaughtered in our presence, after the usual manner, by the butcher, that we might ascertain the quantity of blood lost by the animal, and note the time and manner of its death.

D, Sheep slaughtered in our presence by the butcher.—The blood rushed out in torrents. In one minute after the infliction of the wound the animal became convulsed, and in two minutes died. The quantity of blood lost was exactly thirty-four fluid ounces.

"The heart was found to contain about two drachms of blood. There was nothing remarkable in the other thoracic or abdominal viscera. The sinuses at the basis of the brain were full of blood. The veins on the surface of the brain, cerebellum, and medulla oblongata, were also filled. A fine web of vessels over the corpora striata was beautifully injected with florid blood. A very little serum was found in the ventricles.

"E. In this sheep I first tied both carotids; and, four minutes after, I opened the jugular veins. The blood flowed at first very freely, but afterwards more slowly, unless when the animal was convulsed, when the hemorrhage was constantly observed to increase. In two minutes after the veins had been opened, the breathing became very laboured, and even convulsive. In seven minutes the sheep was powerfully convulsed, and again, and repeatedly afterwards for ten minutes more, the last convulsion observed being at eighteen minutes from the time of opening the jugulars. The blood now flowed slowly and by occasional drops only, and, at twenty-three minutes after the veins had been wounded, the animal died. The quantity of blood lost was thirty-eight fluid ounces.

"The ventricles of the heart were nearly empty, or contained no appreciable quantity of blood. The sinuses of the head were in their usual state; those at the basis of the brain contained less blood than we have hitherto found in them, and the veins on the hemispheres of the brain were less filled; the choroid plexus was pale and empty; the vessels on the basis of the cerebrum were better filled; those ramifying on the basis cerebelli were minutely injected. There was a slight, but very decided, serous effusion within the ventricles." (p. 107—110.)

Various other experiments of a similar kind were tried, but those we have given may be taken as illustrative of the general result, which was, that, although the vascular system of the brain could be less frequently emptied than that of other parts, nevertheless it seems possible to drain it to a very sensible extent; the deficiency of red blood being apparently supplied by the circulation of serum, or by actual effusion. It is worthy of remark, too, that in these experiments the animals required to lose nearly the same quantity of blood to produce death, in whatever manner it was abstracted.

Dr. Kellie conjectures that, if, instead of bleeding animals to death, a small quantity only were abstracted, and the operation frequently repeated, the brain might be still farther drained of its red blood; and goes on to mention some cases in which this phenomenon seems to have resulted from the natural progress of disease. LIEUTAUD relates an instance of this kind, in which a man, about forty-five, had been bled profusely for an acute disease, and who died of syncope. Scarcely a trace of blood could be discovered in the head of this patient.* HALLÉ likewise describes a peculiar disease, which occurred among the workmen in one particular gallery in the coal-mines at Anzain. The whole surface of the body became colourless, together with the inside of the eyelids, lips, and mouth; and in one of them, who died, the blood-vessels in all the cavities were found nearly empty. Even the sinuses of the brain were empty; the choroid plexus was pale, and between two and three scruples of blood were found in the left ventricle. In a case of anæmia, which our author had an opportunity of witnessing, and which is related by Dr. COMBE in the present volume, nearly similar appearances presented themselves. Various examples of an analogous affection are given by some of the continental writers, under the name of anæmia: in one of these the quantity of blood was so extremely small, that the narrator (REISELIUS) concludes his account by the following remark: "Quommodo sanguis qui neque ante neque post mortem effluxit perierit, difficile erit pronunciare."†

These remarks bring to a close the first part of Dr. Kellie's paper; the conclusion derived from his observations being, that the quantity of blood circulating in the head cannot be diminished to any great extent by artificial bleeding; and that, if we

* *Precis de la Medecine Pratique*, p. 72.

† *Miscell. Curios.* Dec. 2, an. 7.

do succeed in getting rid of any sensible portion of red blood, its place is supplied by the circulation of serum, or by its effusion: in short, that we cannot lessen the actual quantity of fluid contained in the cranium, although we may, to a limited extent, alter its nature.

This certainly is a very intelligible assertion, although it may possibly be sometimes overlooked: indeed, when we consider the unyielding nature of the cranium, by which the brain is defended from the pressure of the atmosphere, it is difficult to conceive how any other result could be obtained. The illustration of the late Dr. MUNRO was extremely simple; he used to exhibit in his class a hollow sphere filled with water, and to show that, even when it was inverted, none of the fluid escaped. —Our author conjectured that, if this retention of blood in the head were caused by the cranium guarding it from atmospheric pressure, the simple removal of a portion of the calvarium would produce all the effects which could result from the gravity of the atmosphere; and this investigation commences the second part of his paper.

"To ascertain this point, a portion of the cranium of a dog was removed by the trephine. The dura mater was wounded by the saw, and blood flowed from the surface of the brain. The brain was observed to rise and fall alternately, but so as always to fill the cranium; so that the rise was a sort of protrusion through the opening which had been made. One of the carotid arteries was opened, and in a minute or two afterwards there was an evident gradual sinking of the brain from the margin of the opening. While the blood yet flowed from the carotid, the animal was suspended by the ears, with the view of producing the greatest possible depletion of the vessels of the brain, and allowed to remain in this posture for three hours after death. The brain was sensibly depressed below the cranium, and a space left, which was found capable of containing a tea-spoonful of water. On removing the upper portion of the cranium, the brain appeared of diminished size, or shrunk in its dimensions, so that the membranes, instead of being stretched, seemed loosely extended over it, shrivelled-like and unfilled. The membranes, and the brain itself, were pale and bloodless. No blood was found in any of the sinuses, except at the very terminations of the lateral, at the basis of the cranium. The vessels of the pia mater, ramifying between the convolutions of the brain, were shrunk, and dwindled to the size of small threads. The choroid plexus was also bloodless, and about a drachm and a half of serum was found effused at the basis of the skull.

"Another dog was trepanned with more care, so that a circular portion of bone was removed without wounding the dura mater, which was separated from its adhesion to the cranium for some way round the margin of the opening, by means of a blunt instrument introduced for that purpose. The animal was then bled to death by opening at once the carotid and jugular of one side, suspended by the ears as the former, and examined three hours after death. The brain did not appear so much shrunk within the cranium as in the former dog, but was sensibly depressed. The vessels on its surface were reduced to mere hairs. The brain was remarkably pale, and the choroid plexus bloodless. No blood was found in any of the sinuses, except at the exit of the lateral ones. About a drachm of serum was found at the basis of the brain.

"A third dog was trepanned. The dura mater was slit open, the carotids and jugulars were then wounded, and the animal suspended by the heels. Three hours after death, the lateral sinuses at the basis of the skull contained a good deal of blood. There was a little also within the longitudinal sinus, and about a drachm and a half of blood was found effused between the dura mater and pia mater. The vessels on the surface of the brain were a little more distinct than in the other two dogs, but still very small and bloodless. The substance of the brain, the corpora striata, and choroid plexus, were all very pale. The cerebellum was more vascular and better coloured than the brain. There was a very little serum at the basis.

"Comparing, then, these with the observations made on animals bled to death by simple hemorrhage, it appears that, when the head is entire, the brain still contains a considerable quantity of blood; when previously perforated, very little: the brain continues to fill the cranium in the one case, and subsides within it in the other." (p. 124—126.)

Our readers can judge for themselves of these experiments: to us, we confess, they prove nothing further than what the whole tenor of the previous inquiry had rendered obvious,—viz. that atmospheric pressure, acting on other parts of the body, and not on the brain, must tend to keep this part supplied with blood; while, on the contrary, the pressure being thus artificially admitted, must bring the encephalon into the same condition as the external parts of the body. The position is, that the cranium is always filled, either with solid contents or with fluid, and, consequently, that nothing can be removed without something else supplying its place; nor any thing added without a corresponding displacement of some of the previous contents. This gives rise to the question, whether, by any means, more than the natural quantity of blood can be forced into the brain? In death from hanging and drowning, the respiration is completely interrupted, and the blood accumulates in the lungs and right side of the heart; and, in the former case, the carotids and jugular veins are likewise compressed. Under these circumstances, the vertebral arteries will continue to transmit blood, while its return is nearly, if not altogether, intercepted. Accordingly the external vessels become extremely turgid, and the circumstances are such as would likewise produce a similar condition of the parts within, did they admit of it. VALSALVA and MORGAGNI have entered upon this subject;* and DE HAEN

* *De Causis et Sedibus*, Ep. xix.

hung or drowned fifteen dogs, with a view of determining it; the general result being, that no preternatural congestion could be discovered. These observations agree with the experiments of Mr. Coleman,* as well as with the statement of the present Dr. Munro, by whom, in addition, "particular mention is made of the softness of the brains of criminals." Dr. Kellie had an opportunity of examining the bodies of two pirates who were executed at Leith, and gives the following account of them, so far as relates to the present question.

"On dividing the scalp the blood flowed freely, and in such quantity as to afford ample proof of the congestion of the vessels exterior to the cranium. The dura mater adhered very firmly to the bones, but exhibited no deviation from its usual appearance. All the sinuses contained blood, but in no extraordinary quantity. The larger vessels on the surface, and between the convolutions of the brain, were but moderately filled, and the pia mater was, upon the whole, paler and less vascular than we often find it in ordinary cases. About half an ounce of colourless fluid was found at the basis cranii, and there was some appearance of serosity between the membranes. The texture of the brain was rather soft, but the cineritious and medullary portions of its substance exhibited, as to colour and vascularity, nothing characteristic or remarkable. No sooner was the brain removed, than the blood, yet warm, began to rise and flow profusely from the divided sinuses and vessels at the base of the skull. Rather more than a pound escaped in this way, and afterwards coagulated on the floor." (p. 133.)

This last fact is insisted upon by our author as proving that the blood had been pressing upwards from the congested jugulars and cavæ, without gaining admission into the head till the sinuses were wounded; while, on the other hand, these are sometimes found gorged with blood, although the vessels in which they terminate in the neck be quite empty. On examining the body of a child, "the sinus venosus and cava, and the carotids and jugulars of both sides, were *perfectly empty*. The sinuses of the dura mater were well filled, and the veins of the pia mater ramifying between the convolutions of the brain were *plethoric and congested.*" These facts tend strongly to prove the difficulty either of repleting or depleting the vascular system of the brain.

Our author is of opinion that posture has much less influence than is generally supposed, instancing the cases of professed tumblers, who perform their feats with impunity; and the stooping occupations of many artisans,—the employments of reaping, digging, washing, &c. Two dogs were hung up, one by the ears and the other by the heels; they were poisoned with prussic acid, and examined eighteen hours after death. The external parts of the head were much more congested in the

* Coleman *on Natural and Suspended Respiration.*

latter than in the former; "within the head, the contrast was but trifling."

The next division of our author's paper relates to the connexion subsisting between diseases of the heart and deranged circulation within the head; and in this part of the discussion we are decidedly of opinion, that Dr. Kellie has failed to make good his point,—which is, that "such diseases of the heart have little or no tendency to produce lethargy, palsy, or apoplexy; nor, by consequence, plethora, congestion, or disordered circulation within the head." Only four cases of his own are given in illustration of this opinion; but even these are, in our judgment, decisive against the accuracy of our author's opinion. In the three first the head was not examined; but, in the second of these, one of the symptoms unequivocally evinced the impression made on the brain by the increased force of the heart.

"Mr. G., with a numerous train of distressing symptoms, which too well marked the existence of enlargement of the heart, and of the violent propulsive energy of that viscus, had only one characteristic of any disturbance within the head. On looking upwards to the whitened ceiling of a room, he saw a darkened spectrum, which vanished and reappeared with great regularity. *It was soon discovered that the appearance of this umbra was synchronous with the systole of the heart, so that he used often, in my presence, to count his pulse with the utmost precision, by keeping his eye fixed on the ceiling, and numbering every appearance of the spectrum.* But, independently of this curious symptom, every other function of the brain, during a protracted state of suffering, remained undisturbed till the last moment of his existence." (p. 144.)

In the fourth case, the vessels in the brain were much diseased, and there was some serum effused under the arachnoid; but these are regarded by the author as proving the co-existence of disease in the heart and brain, rather than their mutual dependence.

Dr. Kellie next proceeds to quote numerous authorities to prove that ligatures and tumors, compressing the vessels of the neck, as well as obstructions in the thoracic and abdominal cavities, have little effect upon the circulation within the head, in a sound state of the parts; and concludes his interesting paper in the following words:

"I have thus passed in rapid review the most important of those circumstances, which, from the earliest era of medicine, have been presumed to have a powerful and undoubted tendency to force blood into, and to confine it within, the vessels of the brain, and so to produce a dangerous morbid congestion of that viscus,—circumstances which, accordingly, have very generally been enumerated by systematic physicians as the principal exciting causes of comatose diseases; and, though I am aware of many objections, though I know but too well the unlucky

*pour* and *contre* which embarrass us in almost every subject of medical inquiry, I think a case has been fairly made out, proving that the agency of such causes has been greatly over-rated; that nature has guarded, with peculiar care, the brain and its vessels against such accidents from repletion and depletion, as they must otherwise have been constantly exposed to; and that, while the structure of this organ remains healthy and unchanged, and its vessels sound, those causes are little capable of occasioning plethora, congestion, effusions, or comatose diseases.

"The real causes of apoplexy are changes which take place in the brain itself,—disorganisations and structural alterations of its own texture, and of its vessels and membranes. There are other points, however, connected with the pathology of the brain, and with the causes of the comata which still remain to be scrutinized, and especially the effects of those agents which have a direct and immediate influence on the cerebral functions, such as alcohol, the narcotic poisons, and cold; but the consideration of these is necessarily deferred to some future occasion." (p. 168, 169.)

Before we quit this subject, we must beg, in few words, to remind our junior readers that all this reasoning of Dr. Kellie's may be correct,—that all his positions may be just: it may be true that we cannot force one drop of blood into the head by any increased action of the heart or arteries, nor take one drop away from it by any extent of venesection; yet all this matters not with regard to practice. It is enough for us to know that the blood is propelled towards the head with increased force,—that the fluid, although no more actually enters, may nevertheless exert increased pressure on the brain, and that this may even proceed so far as to burst asunder the sutures, after they have been firmly united,—and that all this may be removed by bleeding. We abstract blood, not to lessen the quantity *in* the brain, but to lessen the quantity pressing *on* the brain; and therefore, in a practical point of view, it matters not one iota whether the opinions, which have now been revived, rather than invented, by Dr. Kellie, be correct or not. We are inclined to think that they are, to a great extent, founded in truth.

*Observations on the Pathology of Scrofulous Diseases, with a View to their Prevention.* By W. P. Alison, M.D. F.R.S.E. and joint Professor of the Theory of Medicine in the University of Edinburgh.

This paper chiefly relates to the time of life at which certain scrofulous complaints are most prevalent,—the circumstances by which they are most liable to be excited,—and their rates of mortality. The author commences by remarking the discrepancy (particularly noticed by Dr. Young,) which prevails among the testimonies borne by medical writers, with regard to the period of life at which pulmonary consumption is most prevalent. The commonly-received opinion that phthisis is scarcely to be met with after thirty-five, does not seem by any means correct. Out of 100 patients who died in one year at La Charité, only 33 were under thirty years of age; 67 were above it, and of these 44 were above forty. Of 135 deaths recorded by Dr. Haygarth, 25 occurred before the age of fifteen, 68 above thirty, and of these 44 were above forty. Of 96 deaths recorded by Dr. Aikin, 36 took place beyond the age of forty-five. Without entering more into individual observations, we may mention that, out of 524 deaths, the records of which are mentioned by Dr. Alison, 94 took place under fifteen; 151 between fifteen and thirty; 279 above thirty, and of these 172, or just one-third, above forty. Of 620 deaths recorded at Berlin, 251 occurred before fifteen; 73 between fifteen and thirty; 296 after thirty, and of these 230 were after forty. The result of these calculations agrees with the author's experience at the Edinburgh New-Town Dispensary, and with our own at a similar and very extensive institution in this metropolis. Indeed, we have long been forcibly impressed with the conviction that fatal disease of the lungs, consisting in purulent expectoration and hectic fever, is much more common in advanced periods of life than is generally supposed. The majority of cases of this kind, which have fallen under our notice, have been in individuals above thirty years of age; and we have seen it in persons above seventy.

Dr. Alison is of opinion that there is a decided difference between the pulmonary consumption which occurs below the age of thirty-five and after that period; the difference consisting in the state of the lungs, rather than in the symptoms. He thinks that simple white tubercles imbedded in the lungs, and having little hardness around them, are much more frequently met with in the young than in the middle-aged or advanced in life; while the ulcerated lungs of the latter is more generally connected with the dark-coloured induration, which we are accustomed to designate by the term *hepatization*. Of twelve persons who died of pulmonary consumption after forty, twelve were examined: in six there were no tubercles, and in the others the number was small. Here again we beg leave to corroborate the accuracy of Dr. Alison's remarks: we have examined the bodies of many persons above forty who have died of this disease, and in these we have very often failed in discovering any tubercles. We have not, indeed, looked for these with the same views which guided our author, but we have looked for them in order to point out to pupils, who were present, the characteristic nature of the pulmonary tubercles; instead of which we have generally found an irregular cavity, of greater or less extent, surrounded by hardened masses, with soft pulpy portions intermixed, easily broken down, and of which, probably, no better idea can easily be given than by saying that the textures were rotten.

"If this observation shall be confirmed by others, it may be supposed that the phthisis which is fatal to young persons, is more generally the effect of organic change of structure,—of the deposition of the matter of the white tubercles,—and depends more on circumstances of predisposition; and that that which is so common in older persons of the lower orders, is more to be ascribed to frequent irritation, and to repeated and neglected inflammation, not originally of an unhealthy character.

"In confirmation of this opinion, we may refer to the observation made by Home and others, that although, in the upper ranks of society, there are more female phthisical patients than male, probably from the greater variation of dress, yet in the lower, among whom the disease is prevalent so much later in life, it is the males that are chiefly subject to it, evidently in consequence of their more frequent exposure to cold and wet.

"Another fact, which still more clearly demonstrates the effect of repeated irritation of the lungs in producing, even in constitutions not predisposed to it, that modification of phthisis which occurs in middle and advanced life, is the well-known unusual frequency of the disease in those workmen who are much exposed to irritation of the lungs, particularly such as are in the constant habit of inhaling various fine powders into their lungs,—in coal-heavers, dressers of flax and feathers, needle-grinders, in the workmen in the mill-stone quarries of Waldshut, and in the stone-masons in this country. I have witnessed many melancholy examples of the disease among the latter class, at the age of forty or more, and in well-made men, of apparently vigorous constitution, and the appearances on dissection have been what I have stated above; and I have reason to believe that there is hardly an instance of a mason, regularly employed in hewing stones in Edinburgh, living free from phthisical symptoms to the age of fifty.

"These facts appear to illustrate the more general fact of the more frequent occurrence of phthisis in the lower ranks than the higher, at advanced periods of life; and to show that the phthisis of that time of life depends chiefly on repeated and neglected irritation and inflammation; and that the means of prevention, or even of cure in cases early and judiciously treated, must be sought chiefly in attention to avoid the ordinary causes of inflammation, and in early and suitable medical treatment when the first symptoms of it appear, such as it is the object of well-devised and well-conducted medical charities to afford." (p. 372—374.)

Regarding the phthisis of earlier periods of life as "almost exclusively a scrofulous disease," our author proceeds to consider, first, the circumstances on which the constitutional predisposition to scrofula depends; and, secondly, the mode in which scrofulous diseases are excited.

Dr. Alison is of opinion that too much attention has been given to the circumstances of hereditary contamination and the influence of climate, and too little to the amount of effect produced by other circumstances. The agency of cold in producing disease is not by any means striking in those most exposed to it, but in those whose constitutions are in such a state as to favour its operation. Thus, persons in the country, exposed to all the vicissitudes of the weather, as they have robuster frames, so they suffer less than the debilitated inhabitants of large towns. Dr. Alison calculates that cold is "intimately concerned in the production of fully two-thirds of the deaths among the lower orders, in a great town in this climate." The application of these remarks is made as follows:

"Now, what is true of the production of disease in general by exposure to cold, seems to be true of the production of scrofulous diseases in particular; but with these limitations:—1. That scrofulous action appears to be excited almost solely in the earlier periods of life; 2. That, to the production of this kind of diseased action, there appears to be required, besides other conditions, a certain peculiarity of habit, not understood, but manifestly hereditary; and 3. That the constitutional debility, which disposes to scrofulous disease from cold, appears to be more permanent and habitual than that which disposes to the other diseases resulting from this cause.

"The term *disease of debility* is much too vague for scientific discussion. It is easy to see that it is not every one who is weakened, even permanently, that becomes thereby disposed to take on scrofulous disease, nor every one who possesses considerable bodily strength that escapes; but if it appear, on careful inquiry, that, of a given number of persons, previously weakened by other causes, a much larger proportion becomes affected with some form of scrofula, than of an equal number, not so weakened, but otherwise similarly circumstanced, we are entitled to conclude that, in many cases, the scrofulous tendency depends in part on, or is much increased by, a state of general debility; and it is probably only in so far as it depends on this cause that it is remediable.

"The facts which seem most decisive as to the connexion of the scrofulous habit with general debilitating causes, may be recapitulated as follows:

"1. The differences in the symptoms and progress of inflammation, when scrofulous and when healthy, appear manifestly to indicate in the former case a languid state of the circulation, particularly in the capillary vessels of the diseased part.

"2. The hereditary disposition to scrofula is chiefly transmitted from parents, and is most observed in children who show evident marks of constitutional debility in other respects.

"3. There is no state of the body, as every practitioner knows, in which scrofulous action is so easily excited, as the state of great and often permanent debility which remains after severe febrile disease, continued fever, small-pox, measles, scarlatina, or which follows the long-continued use of mercury, or accompanies amenorrhœa.

"4. The season at which scrofulous diseases have been observed to prevail most in this climate, is not that when cold weather has recently set in, and is most productive of disease in general, but the end of winter and the spring; and they are then chiefly observed in those young persons who have manifestly lost strength during the continuance of the cold weather. The gradual diminution of strength in weakly persons during cold weather, is naturally to be expected, (conformably with what we know of the agency of cold on the healthy body, and with other physiological principles,) from the habitual chilling of the surface, and more particularly from the little exercise taken by these persons in such weather. It is commonly stated, likewise, that scrofulous diseases are not so prevalent in the coldest climates as in milder, but moister climates, such as that of Britain, where the temperature, during most of the winter and spring, is from 32° to 45°, and the air usually moist; and it is perhaps to be expected that such weather will be more weakening and depressing to the constitutions of young persons than colder, but dry and frosty, weather; because it will restrain them more from taking exercise, and will allow of less evaporation from the surface during any exercise that is taken. The observations made on this head, however, do not seem to me so decisive as they have been thought; because due allowance has not been made for the circumstance of the more northern countries, which have been compared with Britain in this respect, being more thinly peopled, and a much smaller proportion of their population being the inhabitants of large towns.

"5. The most leading fact in regard to the connexion of the scrofulous tendency with debilitating causes, (and one which is of itself sufficient for the most important practical application that can be made of knowledge on this subject,) is the much greater frequency of scrofulous diseases in the inhabitants of great towns, than in the agricultural population of any climate." (p. 380—383.)

"6. I apprehend we may go a step further in pointing out this connexion, and assert, that the weakness of system produced, in many cases, by residence in *hot climates*, during childhood and early youth, gives probably a disposition to scrofula, in those who are afterwards exposed to the exciting causes of it during early life. We know, at least, that a great majority of the inhabitants of these climates, both Negroes and Hindoos, are unusually prone to scrofula when they come to temperate climates, and even suffer from it in some instances in their own, where Europeans are nearly free from it." (p. 397.)

"Lastly, All that has been said of the effect of causes, certainly debilitating, in favouring the tendency to scrofulous diseases, is confirmed by what experience has shown of the effect upon that tendency, and even on the slighter forms of the diseases themselves, of such remedies as are truly and certainly strengthening to the human constitution. We have seen the effect of rural life on the scrofulous habit; and every physician who has practised in a great town can testify that, in circumstances which demand the use of tonic remedies, and of no others, the most powerful that he can prescribe is a residence in the country. 'There,' says Dr. Willan, speaking of the ill-defined symptoms to which he gave the name of simple asthenia, and which he found so common in London, 'a change of pursuits, a more regular plan of diet and exercise, a more clear and purer atmosphere, the salubrious exhalations from growing vegetables, and the grateful stimulus of their odours, in a short time restore vigour to the body, and firmness and serenity to the mind.'" (p. 400, 401.)

After these remarks, which apply to the formation of scrofulous diathesis, the author next proceeds to one of the most important questions connected with the development of strumous disease,—viz. how far tubercles are connected, in their origin and progress, with increased vascular action? The opinions of Bayle, Laennec, Andral, and Dr. Baron, have at various times been laid before our readers; and it is sufficient for us at present to remind them, that these pathologists maintain that the formation of tubercles is quite independent of inflammation. Dr. Alison, on the other hand, has adopted an opposite opinion, and relates various cases in support of his position. The first set of these consists of examples in which the tubercles were not the cause of death, but were found so immediately succeeding the symptoms, and so much connected with the indisputable effects of inflammatory action, that the author regards their formation as having been dependent upon it. The second class of cases occurred in children, (previously unaffected with any pulmonary complaint,) who had been attacked with symptoms of inflammation, which, after lasting some time, had passed away very imperfectly, and the children fallen into consumption, and died within a few months: on dissection, tubercles were found in various stages of progress, but with little, if any, other appearance which could be regarded as the effect of the previous inflammation. One example of each of these is all that our limits will permit us to extract.

"Case I.—J. P., æt. four, of a weak habit, but healthy, was seized, in the beginning of November 1822, with febrile symptoms, head-ache, and pain of the abdomen, attended with tenderness on pressure; which last symptom, however, was not urgent. The febrile symptoms remitted repeatedly, but she continued subject to much pain of the abdomen, generally with a loose state of the bowels, till the beginning of December, at which time I first saw her. The symptoms had just then undergone a remarkable change,—the pain of the abdomen was almost gone, but she had a great deal of vomiting, excited by almost every thing she swallowed, cold extremities, small, often irregular, pulse, and drowsiness approaching to coma, though without head-ache or delirium. These symptoms lasted, with little change, except progressive emaciation, for ten days; the vomiting then ceased, and she was seized with convulsions, followed by coma and dilated pupils; in which state she lay four days, with occasional returns of convulsion, and died on the 19th December.

"On dissection, the ventricles of the brain were found distended with serum.

"Almost the whole peritoneum, except the covering of the stomach, was covered with an exudation, resembling the coagulable lymph so often thrown out on it by inflammation; except that it was generally of a somewhat yellower colour, and was disposed chiefly, though not entirely, in the form of round tubercular eminences, of various sizes, none so large as a split-pea. By the intervention of this matter, numerous adhesions of the liver to the parietes of the abdomen, and bowels to each other, had taken place. In various places, where the exudation was thickest, the peritoneum was thickened and very vascular. In the pelvis there was an irregular mass, of the same appearance, which hardly adhered to the peritoneum. The ligamenta lata of the uterus were much thickened, and of a deep red colour, and, on cutting into them, much thick pus was found between their layers.

"This was manifestly a case of conversion of abdominal disease into hydrocephalus. Had it not been for that conversion, the inflammatory appearances in the abdomen might probably have subsided, the pain of the abdomen having almost ceased long before death. The disease would then, I have no doubt, from what I have seen in other cases, in which there were depositions on the peritoneum in precisely similar forms, but of larger size and somewhat firmer consistence, have passed into the form of the tuberculated accretions on the peritoneum described by Dr. Baron; and, if this had been fatal at a subsequent period, it would have been impossible to judge whether inflammation had produced the tubercular masses that were found or not; a point, on which the appearances observed in this early stage of the disease in the present case left, I believe, no doubt whatever in the minds of the gentlemen who witnessed this dissection. (p. 411—413.)

"Case VI.—J. S., æt. five, a boy of ordinary stature, and pretty stout, but somewhat ricketty, and with a small scrofulous sore on his leg, was attacked, in the end of November 1815, with well-marked pneumonic symptoms. While these were recent, he was seen by different medical men, who had no doubt of their nature, and he was bled twice at the arm, and used the other usual remedies, with very imperfect success; the heat of skin, febrile oppression, and dyspnœa, abated somewhat; but his breathing continued short, his cough very troublesome and dry; and he passed gradually into the state of perfect hectic, the rigors in the afternoons and morning-sweats being unusually severe. He died, considerably emaciated, in the end of January 1816.

"On dissection, the lungs were found of the natural spongy texture throughout, and the disease appeared to have been confined to the bronchial glands, which were enormously enlarged, and all converted into the usual cheesy or tubercular matter. There was no other disease in the thorax or abdomen." (p. 425, 426.)

If it be argued that the tubercles found after death in these cases were not produced by the inflammation, but that this only accelerated their development, Dr. Alison replies, that it is of little practical importance whether we regard the inflammation as capable of generating these bodies, provided we concede to it the power of thus accelerating their fatal progress. He regards this excitation of tubercles by inflammatory action as placing a practical limit to the use of tonics, and urges the employment of all means to counteract the tendency to scrofula, "*while it is still safe to use these means with effect.*"

---

### DIVISION II.

### FOREIGN.

ART. IV.—*Leçons sur les Epidemies et l'Hygiene publique.* Par F. E. FODERÉ. Tom. II.—pp. 565. Paris, 1823.

[Concluded from page 340.]

THE *third order* of diseases, or those arising either from a variation in the temperature, or in the moist or dry state of the atmosphere, necessarily includes a great number of diseases not usually considered as epidemics, but which the arrangement of our author compels him to notice.

The first chapter is a very long one, but it contains an enormous mass of matter: it commences with a description of the inflammatory fever, or synochus, and extends to the consideration of the inflammation of almost all the different organs of the body, including the brain, the throat, the pleura, the intestines, &c. M. Foderé we are glad to find boldly advocating the use of the lancet in these affections: and we shall quote the following case, which he has given at page 340, with the very judicious animadversions he has made on the practice therein adopted.

A soldier, aged twenty-four, of a sanguine temperament, was admitted into the Hospital Val de Grace, on the 4th February, 1821, having been six days ill in barracks. The following were the symptoms he was labouring under: a most violent pain in the head, the eyes injected and expressing dejection, countenance flushed, tongue very red at the point and at the edges, great thirst, pain at the stomach; contracted pulse, very small and frequent; a dry and extremely hot skin, pains in the loins and legs, and constipation.—Thirty leeches to the epigastrium, low diet, and lemonade.

5th.—The symptoms undiminished. Thirty more leeches.

6th.—In the same state. Twenty leeches.

7th.—The tongue less red; not so much pain in the epigastrium; diarrhœa. Ten leeches to the anus.

8th.—Diminution of the diarrhœa; delirium, faintings. Sixteen leeches in the course of the jugulars, and sinapisms to the feet.

9th.—The same symptoms. Sixteen leeches to the mastoid apophyses, and sinapisms to the legs.

From the 10th to the 12th, less delirium, but the other symptoms the same; and, after the 12th, prostration of strength, cough, spasms, delirium and continual moanings, involuntary stools: in short, death on the 24th of the same month.

On opening the head, the arachnoid and pia mater are found strongly injected with blood; false membranes formed; remarkable hardness of the brain, and serum in the ventricles. In the chest, the trachea and bronchiæ red; in the larynx, two small ulcerations; the lungs gorged with blood; the pleura thickened and inflamed, &c. In the abdomen, some few ulcerations in the jejunum and ileum; the stomach in general sound, with some minor diseased appearances not worth extracting.

This case M. Foderé quotes from the Journal of Medicine. The author, M. Gasc, denominates this to be a gastro-enteritis in the highest degree, accompanied with a remarkable inflammatory diathesis. According to him, the inflammation began in the stomach, then went to the intestines, from thence to the head, and from the head to the chest: in short, he adds, it was beyond the *activity of our art.* "Yes," says M. Foderé, "beyond the activity of our art in its infancy: yes, beyond the reach of 120 leeches; but it would not have been beyond the reach of general blood-letting employed early."

But we need not pursue these reflections, since we are confident that they are not needed on this side of the Channel; but, on the contrary, the love of bleeding, and the boldness with which it is occasionally practised, require perhaps rather a check than a recommendation. There is also great truth, we apprehend, in the caution which our author gives against the use of drastic purgatives, in the inflammatory affections of the chest especially. Upon the whole, this long chapter well deserves an attentive perusal; though, what some of the diseases of which he treats have to do with epidemics or public hygiene, we are at a loss to conceive.

Chapter the second treats of *bilious and ardent bilious fever.* The cause of these fevers has been attributed by many writers to saburræ in the primæ viæ; but to this M. Foderé replies, that the bilious fever in the South of France and in Italy frequently attacks those who are remarkably temperate in their diet; though, without doubt, he adds, excesses of any kind will render this disease more intense. We find nothing in the description of this fever to induce us to extend our extracts. The occurrence of erysipelas of the head and face, and the occasional change of the disease to the intermittent type, are noticed; and the ardent bilious fever, a description of which will be found at page 363, is in fact only a severer form of the former malady, with the inflammatory symptoms at the commencement of the attack more strongly marked. This last variety of bilious fever is the common disease of hot countries, and is often met with raging epidemically: nevertheless, observes M. Foderé, this fever is frequently met with in all countries during the dry and hot season of the year. Passing over a page or two, in which the prevalence of a bilious constitution in certain countries and individuals is asserted, we find our author pointing out the abuse of venery as a very frequent predisposing cause of this class of maladies.

On the subject of treatment, the first remark we meet with, demanding our notice, is that the bilious fever, however simple, is never without a tendency to inflammatory action, and that, therefore, the treatment must be always antiphlogistic; but he looks upon the notion of the alteration of the quality, and excess in the quantity, of bile as being the causes of the fever, as highly erroneous; for, he adds, we may, by means of drastic purgatives, produce the evacuation of the same quantity of bile in a state of health: and with respect to its alteration, that takes place only at the moment it is discharged, and is frequently the immediate consequence, either of an affection of the mind, of dentition, a slight derangement of the system, or the disagreement of some article of diet; and, from these and other considerations, M. Foderé is inclined to look upon the state of the bile not as the cause, but the effect, of the fever; and thus the name we have given rather belongs to one of the phenomena than describes the real nature of the complaint, the essence of which he believes to consist in irritation of the hepatic organs, a point which he argues at some length, and which our contracted limits will not permit us to dwell upon; and therefore we proceed to give our author's indications of cure, which are two. The first is to temper the ardent heat of the patient, arising from the absorption of the biliary particles, the irritation of the secreting organ of the bile and its reservoir, &c.; and the second (which unfortunately, he says, is too often placed in the first rank,) consists in evacuating the bile already secreted, and become a mass of irritating matter in the intestinal canal. Our author's practice being founded upon this view of the disease, we may easily understand that he is an enemy to violent purgatives or emetics in the first attack: he employs bleeding, general or local, according to the constitution, age, &c. of the patient, and lays great stress upon emollient drinks, fomentations, the warm bath, and a strict abstinence from all nourishment, either solid or liquid, especially wine and coffee, and advocates a perpetual renewal of the air of the apartment; and then, he observes, should nausea and bitterness of the mouth, with a loaded state of the tongue, continue after twenty-four or forty-eight hours, recourse may be fairly had to emetic, the *timely* administration of which he highly praises.

The remaining remarks of this chapter do not appear to us either novel or interesting: the use of proper laxatives, the propriety of administering the bark after a remission of the febrile symptoms, is discussed; all of which are familiar to our readers.

The *cholera morbus* of Europe and the Indies, and the different species of *colic*, form the comprehensive subject of the third chapter of this division; but we shall do no more than give the title. The account our author gives of the cholera of India is meagre: it is evident that he is not acquainted with the best and latest English accounts of the disease; and his short descriptions of the cholera morbus of Europe, of the colica pictonum, and other varieties of these affections, do not afford any novelty, nor add any thing to our present stock of knowledge. We, therefore, proceed to the consideration of *simple catarrhal fevers*, which is the subject of Chapter ivth; and a few words of commentary will suffice for this class of diseases.

M. Foderé is of opinion that these epidemic visitations are not contagious; and that, although the mucous membranes of the nose, throat, &c. appear to suffer most, the stomach is the part primarily affected; and he remarks the extreme difference, with regard to fatality, which these epidemics exhibit at different times. The most striking example, which he quotes from Charles Frederick Lœw, is that, in the catarrhal fever at Vienna in 1729, although 60,000 people suffered the attack of the disease, but few died; whereas in 1730, at the same place, 6,493 persons died in consequence of the re-appearance of the same disease. With regard to the symptoms and treatment of this malady, many pertinent observations are made; but they are familiar to most of our readers, and we cannot spare room for their insertion. Indeed, we feel the necessity of being select in our quotations stronger than ever, for the third volume of this work has just reached us, and in the first page we find it announced that our author, being unable to finish all he has to say within the limits of his original design, has a *fourth* volume in the press, and which will speedily appear. Under these circumstances, our readers will surely rather thank us for passing over these chapters, which, however well written or abounding in judicious directions, offer no striking novelty either of facts or doctrines; since it has appeared to us that, although decidedly superior to most modern continental works, it is not one from which our countrymen can profit very greatly; that is, as far as relates to the mode of treatment of the numerous diseases of which our author treats. For this reason we shall entirely omit all notice of the fifth chapter, which treats of *pituitous, mucous,* and *mesenteric fevers,* and proceed to give a few extracts from the sixth chapter, entitled of *pulmonary catarrh* and *catarrhal phthisis.*

Our author, in the beginning of this chapter, draws a frightful, but we fear a just, picture of the ravages caused by pulmonary complaints throughout the most favoured countries of Europe; and he says that, in those departments of France which he annually visits, the pulmonary phthisis is every where looked upon as the principal source of mortality. In the *South of France, upon the borders of the Mediterranean*, it commits the most horrible ravages; and yet, with this truth unequivocally avowed by natives of the country, we still continue, with an obstinate adherence to antiquated prejudices, to consign our consumptive invalids to a tedious journey, and a residence where they are deprived of many of the comforts which habit has rendered almost essential to their existence, and where the disease which is exhausting them is met with as frequently as in England. It appears that, out of 62,447 deaths, which took place in Paris in the years 1816, 1817, and 1818, thirteen thousand eight hundred and eighteen fell victims to various diseases of the chest. We need not pursue this part of the subject further.

The object, however, that M. Foderé appears to have principally in view in this chapter, is to draw a distinction between the catarrhal phthisis, the tubercular, and the scrofulous. We do not think that he establishes his point at all satisfactorily; his reasoning appears to us fanciful, his illustrations no less so; and his concluding remark, that the same causes produce the one or the other species of consumption, and that the same considerations apply equally to every possible case of pulmonary phthisis, renders all argument upon the subject useless.

In describing the disease which we call *a cold*, M. Foderé remarks upon the singular alteration in the secretion from the mucous membrane of the nose and fauces, from the commencement of the attack: at first, he remarks, it is so caustic as to excoriate the upper lip, the nostrils, &c.; and in the second period its nature is entirely changed, without any apparent alteration of the secreting surface.

Our author, after describing one or two epidemic catarrhs, next proceeds to draw his line of distinction between catarrhal and tubercular phthisis; but into this we shall not follow him. We think he here refines too much, especially when he says that, in tubercular phthisis, the first symptom that gives alarm to the relatives is *almost always* a spitting of blood. Now we are decidedly of opinion that a common catarrh is much oftener the cause of tubercular consumption, and more frequently brings the latent disposition into action, than any accidental rupture of a blood-vessel: nor can we agree with him that the catarrhal consumption is never preceded by this phenomenon. Among the causes of this malady, the only one which M. Foderé insists upon in a manner peculiarly strong, is the practice of *onanism;* and we are of opinion that his remark is just, and no less important than true. The guardians of the youth of both sexes should bear this constantly in mind; it is a vice that commences at a much tenderer age than is commonly imagined, and which due vigilance may prevent.

There is nothing particularly attractive in M. Foderé's remarks upon the prognosis of consumption; and his method of treatment does not differ materially from that generally pursued. We find that a Tyrolese practitioner, of the name of Salvadori, in the latter part of the last century, founding his system upon certain passages in the works of Hippocrates, recommended drinking wine, eating ham and other salted meats, and using strong exercise, as the best remedies in consumption; and our readers will observe that this plan is not materially different from a mode of treatment which has lately been fashionable in our own country. M. Foderé justly condemns this indiscriminate recommendation; but admits, what indeed is undeniable, that there are certain cases of phthisis which require nutritious diet and strong exercise.

Our author proceeds next to say, "Since colds produce such fatal effects, what ought we not to do to preserve ourselves from them?" and this leads him to enumerate several precautionary methods, but which, he justly observes, cannot be followed by the mass of mankind.

The chapter concludes with some sensible general remarks upon the mode of bringing up children who may be suspected of inheriting a hereditary disposition to this disease; and some particular cautions relative to the prevalence of the vice of onanism, and the most efficacious method of preventing this detestable and ruinous practice.

We have now gone through our author's second volume, and have only to lament that the rapidity with which this work has been published has compelled us to comprise our review of a volume within very brief limits, otherwise we should scarcely have an opportunity of noticing any contemporary work.

